# Pleomorphic adenoma gene1 in reproduction and implication for embryonic survival in cattle: a review

**DOI:** 10.1093/jas/skae103

**Published:** 2024-04-08

**Authors:** Michael J D’Occhio, Giuseppe Campanile, Pietro S Baruselli, Laercio R Porto Neto, Ben J Hayes, Alf Collins Snr, Marina R S Fortes

**Affiliations:** School of Life and Environmental Sciences, Faculty of Science, The University of Sydney, Sydney, NSW, Australia; Department of Veterinary Medicine and Animal Production, University of Naples Federico II, Naples, Italy; Faculty of Veterinary Medicine and Animal Science, Department of Animal Reproduction, University of Sao Paulo, Sao Paulo, Brazil; CSIRO, Agriculture and Food, Brisbane, QLD, Australia; Queensland Alliance for Agriculture and Food Innovation, The University of Queensland, Brisbane, QLD, Australia; CBV Brahman, Marlborough, Central Queensland, QLD, Australia; School of Chemistry and Molecular Biosciences, The University of Queensland, Brisbane, QLD, Australia

**Keywords:** cattle, embryo, pleomorphic adenoma gene, *PLAG1*

## Abstract

The pleomorphic adenoma gene1 (*PLAG1*) encodes a DNA-binding, C_2_H_2_ zinc-finger protein which acts as a transcription factor that regulates the expression of diverse genes across different organs and tissues; hence, the name pleomorphic. Rearrangements of the *PLAG1* gene, and/or overexpression, are associated with benign tumors and cancers in a variety of tissues. This is best described for pleomorphic adenoma of the salivary glands in humans. The most notable expression of *PLAG1* occurs during embryonic and fetal development, with lesser expression after birth. Evidence has accumulated of a role for PLAG1 protein in normal early embryonic development and placentation in mammals. PLAG1 protein influences the expression of the *ike growth factor 2 (IGF2)* gene and production of IGF2 protein. IGF2 is an important mitogen in ovarian follicles/oocytes, embryos, and fetuses. The *PLAG1-IGF2* axis, therefore, provides one pathway whereby PLAG1 protein can influence embryonic survival and pregnancy. PLAG1 also influences over 1,000 other genes in embryos including those associated with ribosomal assembly and proteins. Brahman (*Bos indicus*) heifers homozygous for the *PLAG1* variant, rs109815800 (G > T), show greater fertility than contemporary heifers with either one, or no copy, of the variant. Greater fertility in heifers homozygous for rs109815800 could be the result of early puberty and/or greater embryonic survival. The present review first looks at the broader roles of the *PLAG1* gene and PLAG1 protein and then focuses on the emerging role of *PLAG1*/PLAG1 in embryonic development and pregnancy. A deeper understanding of factors which influence embryonic development is required for the next transformational increase in embryonic survival and successful pregnancy for both in vivo and in vitro derived embryos in cattle.

## Teaser

This review explores how the pleomorphic adenoma gene1 (*PLAG1*) which is associated with cancers may also be fundamentally important in embryonic development and the establishment of pregnancy in mammals.

## Introduction

The major cause of reproductive loss in cattle is the failure of embryos to progress to implantation and pregnancy. Fertilization rates in both beef and dairy cattle are in the order of 85% to 100%; however, only 40% to 60% of embryos establish a pregnancy (Diskin et al., 2016; Lockhart et al., 2023). In recent reviews, we have argued that the next transformational change in reproductive efficiency will require a deeper understanding of the biology of early embryo development in cattle (D’Occhio et al., 2019b, 2020a, b; Campanile et al., 2021). This applies to both natural mating and assisted reproduction. A critically important feature of early embryo development is the dialogue between embryo and uterus in the period before embryo attachment and during implantation (Hantak et al., 2014; Rizos et al., 2017; Sponchiado et al., 2017, 2019, 2020; Aguilera et al., 2022; Binelli et al., 2022; Cajas et al., 2022; Tesfaye et al., 2022). Factors involved in embryo-uterine communication include the transforming ß superfamily (D’Occhio et al., 2020a), cell-cell adhesion molecules (D’Occhio et al., 2019b), kisspeptin (D’Occhio et al., 2020b) and immune factors (Campanile et al., 2021), among others. Our reviews, and those of others, have noted the complexity of events associated with early embryo development, attachment of the conceptus to the uterine epithelium, and implantation. The reviews have identified major gaps in our understanding of early embryo development in cattle. The gaps in knowledge largely explain the relatively modest progress over the past 40 yr in reducing high embryo loss in cattle. High embryo loss applies to both in vivo and in vitro derived embryos. Embryo loss is comparable after natural mating, artificial insemination, or embryo transfer (Hansen, 2020). The transfer of a bovine embryo to a recipient at day 7 of development avoids the relatively large loss of embryos that occurs in the first 7 d after fertilization. However, there is still considerable loss between the transfer on day 7, and day 21, when embryo attachment has commenced (Hansen, 2020). Therefore, the transfer of a bovine embryo on day 7 of early development does not overcome all the embryo losses in cattle that occur before implantation.

**Figure 1.**
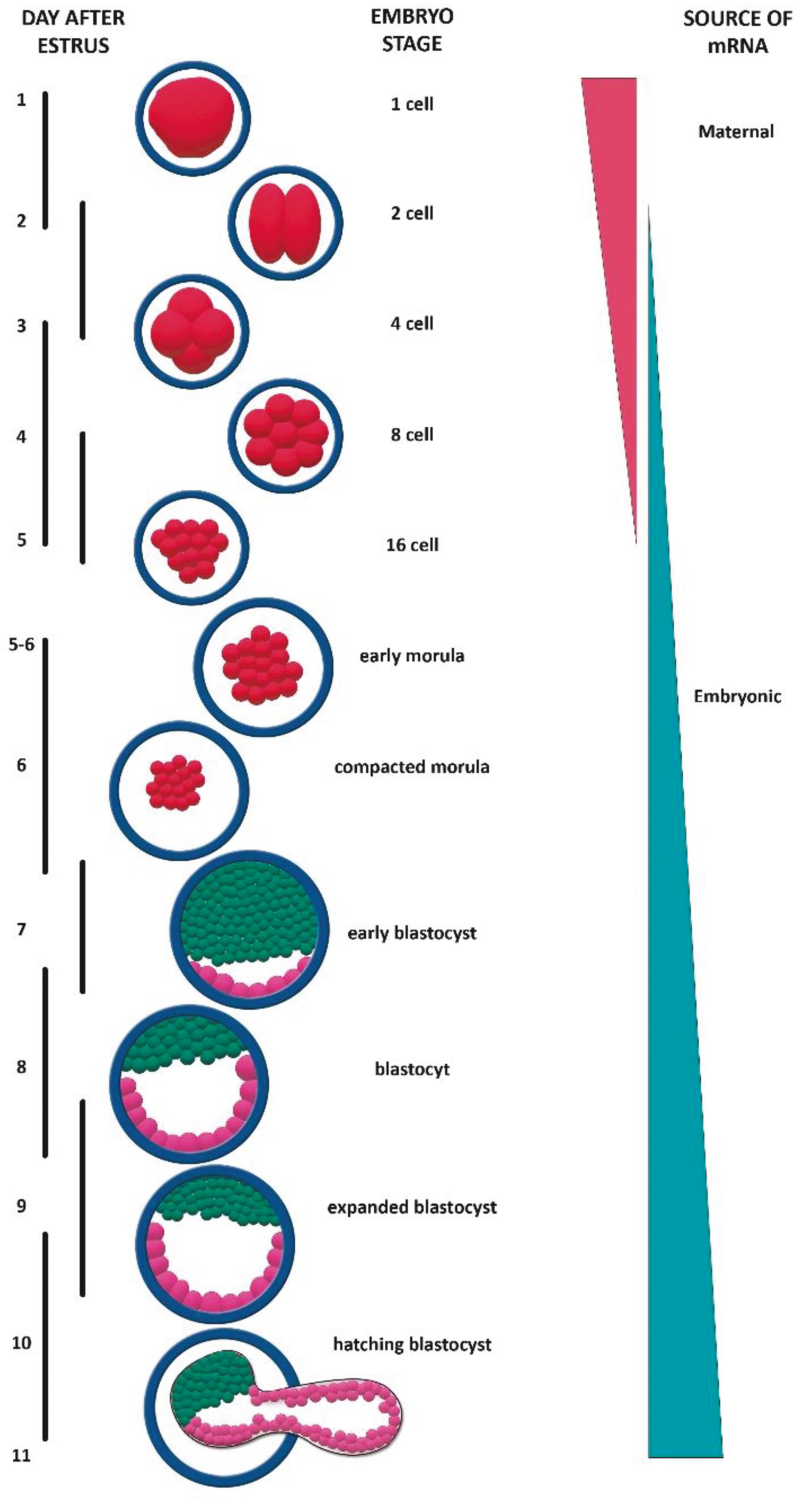
Zygotic genome activation in cattle. *PLAG1* is maternally imprinted and PLAG1 protein derived from paternally expressed *PLAG1* could potentially be present in embryos from the 2 to 4 cell stage.

The present review seeks to build on our earlier articles and looks at the potential role of the pleomorphic adenoma gene1 (*PLAG1*) in early embryonic development. The *PLAG1* gene encodes a DNA-binding, C_2_H_2_ zinc-finger protein which acts as a transcription factor that regulates the expression of diverse genes across different organs and tissues (Voz et al., 2004; Abdollahi, 2007; Wagner and Zhang, 2011; Adnani et al., 2018). The most notable expression of *PLAG1* occurs during embryonic and fetal development with lesser expression after birth (Hensen et al., 2004; Tang et al., 2013; Habib et al., 2018; Madissoon et al., 2019; Li et al., 2020a). There is a paucity of information on the transcriptional regulation of the *PLAG1* gene. The neurogenic factor Hmga2 induces expression of *PLAG1* in neuronal progenitor cells (Sakai et al., 2019) while microRNA-141 shows translational regulation of *PLAG1* mRNA (Tang et al., 2013). In early embryos, PLAG1 protein was reported to act at conserved Alu/B1 elements in the promotor region of over 1,000 genes associated with ribosomal assembly and protein synthesis (Madissoon et al., 2019). Rearrangements of the *PLAG1* gene, and/or overexpression, are associated with benign tumors and neoplasia in different tissues (Matsuyama et al., 2011). This is best described for pleomorphic adenomas of the salivary glands in humans, which gave the gene its name (Voz et al., 1998, 2000; Åström et al., 1999; Debiec-Rychter et al., 2001; Hensen et al., 2002; Declercq et al., 2005; Asp et al., 2006; Van Dyck et al., 2007; Skálová et al., 2021). There is evidence of a role for PLAG1 protein in normal early embryonic development and placentation. In mice, oocytes with low amounts of maternal *PLAG1* transcripts showed a delay in zygotic genome activation, and 2-cell-stage embryonic development (Madissoon et al., 2019). The *PLAG1* gene is maternally imprinted and an ongoing role for PLAG1 protein during embryonic development may depend on the expression of paternal *PLAG1* (Moore & Haig, 1991; O’Doherty et al., 2012; Barlow & Bartolomei, 2014; Plasschaert & Bartolomei, 2014; Adhami et al., 2015; Jiang et al., 2015; Lafontaine et al., 2020). In cattle, minor activation of the embryonic genome occurs at the 2-cell embryo stage, with major activation at the 4- to 8-cell stage (Telford et al., 1990; Memili et al., 1998; Memili & First, 1999, 2000; Dean et al., 2001; Kaňka et al., 2003; Meirelles et al., 2004; Ruddock et al., 2004; Gad et al., 2012; Ozawa et al., 2012; Graf et al., 2014a, b; O’Doherty et al., 2015; Jukam et al., 2017; Jiang et al., 2018; Lavagi et al., 2018; Duan et al., 2019; Halstead et al., 2020; Ivanova et al., 2020; Figure 1). *PLAG1* is polymorphic in cattle and any potential action of PLAG1 protein on ongoing embryonic development may depend on the nature of the paternal *PLAG1* allele. *PLAG1* can influence the production of ike growth factor 2 (IGF2), H19, leukemia inhibitory factor (LIF), ß-catenin, and cytokines. These factors are all variously associated with embryonic development, uterine attachment, and implantation (Niemann & Wrenzycki, 1999; Han et al., 2003; Gabory et al., 2009; Agrogiannis et al., 2014; Jiang et al., 2015; Smith et al., 2015; Sferruzzi-Perri et al, 2017; Campanile et al., 2021; Llobat, 2021; Willhelm et al., 2021; Zhou et al., 2021; Sandovici et al., 2022). The role of LIF and other cytokines, and the LIF receptor, in embryonic development and implantation is comprehensively discussed in earlier reviews which are complemented by the present review (Guzeloglu-Kayisli et al., 2009; Robertson et al., 2018; Campanile et al., 2021; Namiki et al., 2023). The role of catenins during early vertebrate development through cell adhesion in association with cadherins (Stepniak et al, 2009; D’Occhio et al., 2019b) and intracellular signaling in the Wnt/β-catenin pathway (Valenta et al., 2012; Liu et al., 2022) also have been well documented. In cattle, polymorphisms of the *PLAG1* gene are linked with fetal and postnatal growth and adult phenotypes including fertility (PLAG1 and Phenotype in Cattle below).

The approach adopted in the present review is to first provide a general background on the *PLAG1* gene and PLAG1 protein. We then consider relationships between *PLAG1* polymorphisms and phenotypes in cattle. This is followed by a focus on the role of *PLAG1*/PLAG1 in early embryonic development. In keeping with our earlier reviews, this review seeks to build awareness of the complex biology of embryonic development. Our consistent argument has been that a deeper understanding is needed of the factors that impact early embryo development before a meaningful transformational change can be made in the efficiency of both natural mating and assisted reproduction in cattle.

## Discovery of *PLAG1* Gene and PLAG1 Protein

The *PLAG1* gene and PLAG1 protein were described from 1997 to 1998 (Table 1). The seminal report showed the *PLAG1* gene to be associated with a chromosome translocation at 8q12 that was linked with pleomorphic adenomas of the salivary glands in humans (Kas et al. 1997a, b). The same laboratory described two related human proteins, PLAGL1 and PLAGL2. The protein PLAGL2 also binds to DNA and has similar properties as PLAG1 protein (Kas et al. 1998). The *PLAG1*/PLAG1 family members were subsequently assigned various names based on the association of *PLAG1* mutations with different phenotypes in different species (Table 1). In the absence of *PLAG1* gene rearrangement, and/or overexpression of *PLAG1*, PLAG1 protein can have antiproliferative activity and tumor suppression. Hence, the regulated expression of *PLAG1* is associated with normal cellular function in different tissues, while overexpression is linked with benign tumors and malignancies (Zatkova et al., 2004). Overexpression of *PLAG1* leads to overproduction of PLAG1, rather than changes in the structure of PLAG1 protein. PLAG1 stimulates the *IGF2* gene and excess production of IGF2 is considered one mechanism linked to tumors and cancers (Voz et al., 2000, 2004; Zatkova et al., 2004; Akhtar et al., 2012).

**Table 1.** Discovery of the pleomorphic adenoma gene (*PLAG1*) family members

Name	Function described	Year	Species	Reference
*PLAG1*: pleomorphic adenoma gene	Activation in salivary gland tumorigenesis	1997	Human	Kas et al. 1997a, b
^*^LOT1: lost-on-transformation	Decreased or lost expression in transformed ovarian epithelia cells that developed into malignant ovarian tumors	1997	Rat	Abdollahi et al. 1997a, b; see also Abdollahi 2007
^*^ZAC/ ZAC1: zinc-finger protein found to regulate apoptosis and cell cycle arrest	Induction of apoptosis and G_1_ cell cycle arrest and inhibition of tumor growth	1997	Mouse	Spengler et al. 1997
^*^PLAGL1: PLAG1 like zinc-finger 1 PLAGL2: PLAG1 like zinc-finger protein 2	Identified by screening mouse embryo and human fetal kidney cDNA libraries using PLAG1 open reading frames (ORF)	1998	Human, Mouse	Kas et al. 1998

^*^Same *PLAG*/PLAG family member.

**Figure 2.**
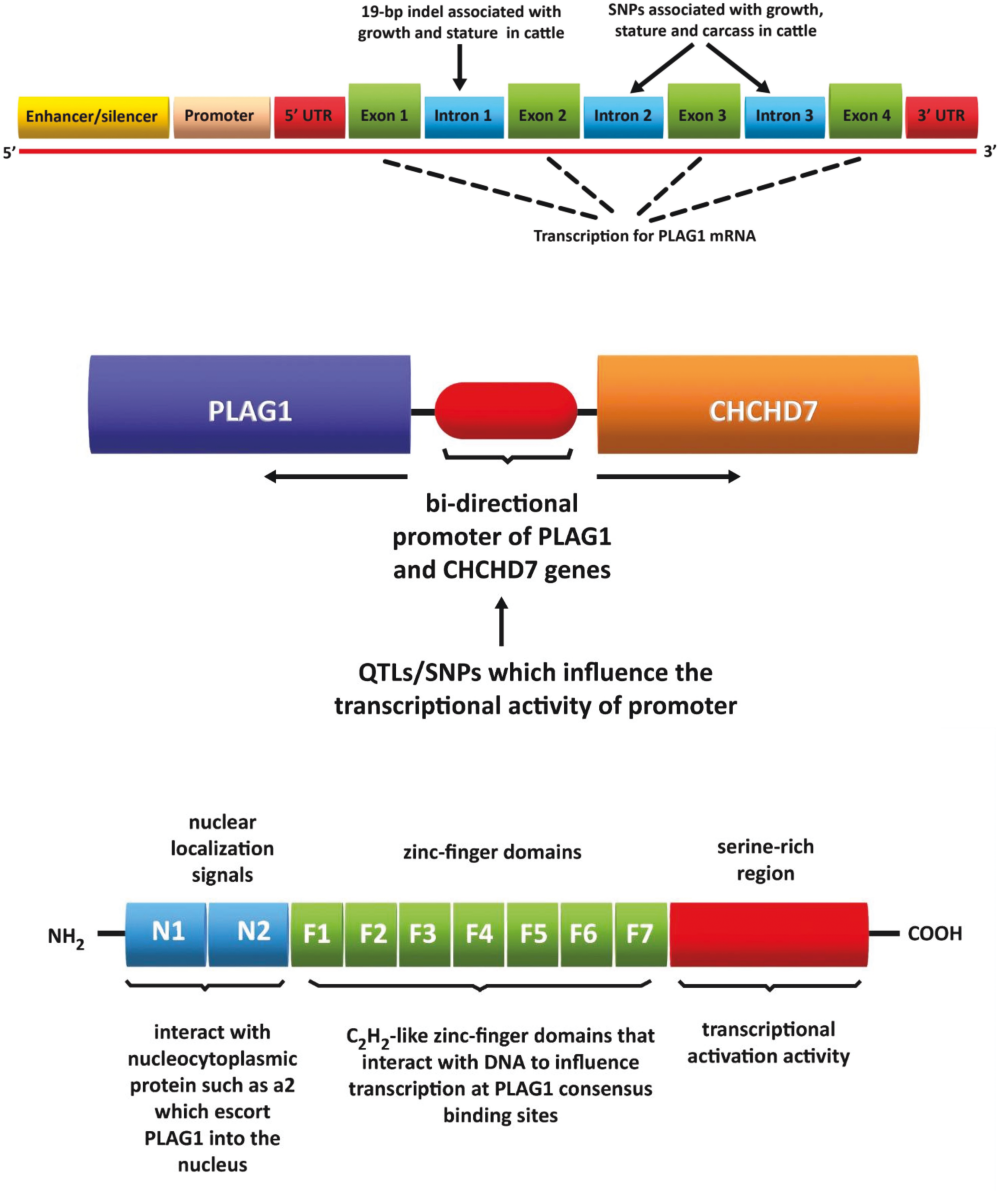
The putative structure of the *PLAG1* gene in cattle and variants of PLAG1 associated with different phenotypes. Indel, insertion/deletion; SNPs, single-nucleotide polymorphisms (top); the common bi-directional promoter of the *PLAG1* and *CHCHD7* genes. QTLs/SNPs in the promoter influence the transcriptional activity of *PLAG1* and *CHCHD7* and phenotypes in cattle including growth and stature. QTLs, quantitative trait loci; SNPs, single-nucleotide polymorphisms (middle); and the structure of PLAG1 protein and domains associated with translocation to the nucleus and binding to DNA. PLAG1 typically binds to the promoter of target genes to influence transcription (bottom).

In humans, the *PLAG1* gene comprises 6 exons and 5 introns. *PLAG1* has yet to be fully described in cattle and is presently thought to comprise 3 introns and 4 exons (Van Dyck et al., 2007; Figure 2). In cattle, a 19-base pair insertion/deletion (19-bp indel) at Exon 1, and single-nucleotide polymorphisms at Exons 3 and 4, are associated with growth, stature, and carcass traits (Karim et al. 2011; Littlejohn et al. 2011; Zhong et al., 2019; Figure 2). PLAG1 mutations were also associated with age at puberty and circulating levels of IGF1 in heifers (Fortes et al., 2013). *PLAG1* is located within the same quantitative trait loci as the coiled-coil-helix-coiled-coil-helix domain containing 7 (*CHCHD7*) gene, which is also associated with growth and stature in several species including cattle (Li et al., 2020a; Xu et al., 2020). Both genes share the same bi-directional promoter and SNPs known to influence the transcriptional activity of the promoter impact the expression of *PLAG1* and *CHCHD7* (Karim et al., 2011; Fink et al., 2017; Figure 2). PLAG1 protein is comprised of three regions with distinct functions: a region with nuclear translocation signals for the transfer of PLAG1 to the nucleus; C_2_H_2_-like zinc-finger domains that interact with DNA to influence transcription; a serine-rich region that has transcriptional activation activity (Braem et al., 2002; Hensen et al., 2002; Figure 2).

## *PLAG1* and Phenotype in Cattle

The most studied relationships between *PLAG1*/PLAG1 and phenotype in cattle are for growth and stature (Karim, et al., 2011; Pryce et al., 2011; Visscher & Goddard, 2011; Boitard et al., 2016; Takasuga, 2016; Taye et al., 2017; Utsunomiya et al., 2017; Bouwman et al., 2018). As noted above, *PLAG1* is most noticeably expressed during fetal development and *PLAG1* polymorphisms are linked with differences in birth weight and calving ease in cattle (Littlejohn et al., 2011; Pausch et al., 2011; Utsunomiya et al., 2013). *PLAG1* polymorphisms are also associated with growth, mature body size, and stature, in different breeds of cattle including Holstein-Friesian (Littlejohn et al., 2011; Zhao et al., 2015), Holstein Friesian × Jersey (Karim et al., 2011), Chinese (Xu et al., 2018; Hou et al., 2019; Zhong et al., 2019; Zhou et al., 2019; Li et al., 2020b), European (Randhawa et al., 2015; Zhao et al., 2015), African (Randhawa et al., 2015), and Japanese Black (Hoshiba et al., 2013; Sasaki et al., 2013). Other commercially important production traits in cattle linked with *PLAG1* polymorphisms are carcass weight and meat yield (Nishimura et al., 2012; Hoshiba et al., 2013; Bolormaa et al., 2015; Song et al., 2016; Hay & Roberts, 2018; Zhang et al., 2019), milk quality (Zhao et al., 2015; Fink et al., 2017), and adaptation (Porto-Neto et al., 2014; Boitard et al., 2016). *PLAG1* influences growth and production traits in goats (Wei et al., 2021) and sheep (Wu et al., 2019; Pan et al., 2022).

**Figure 3.**
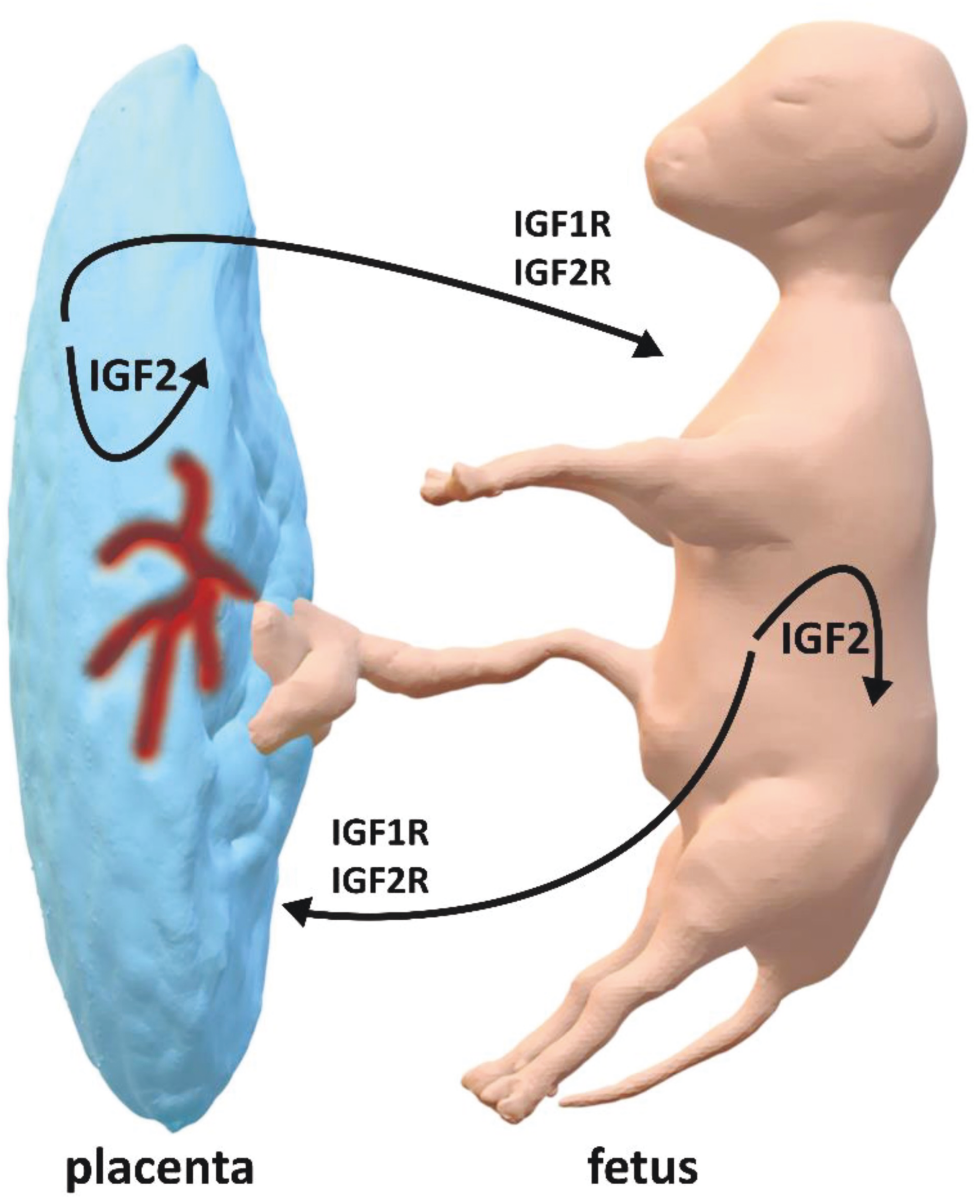
Insulin-like growth factor 2 (IGF2) is produced by the fetus and placenta and has both local and reciprocal action between the fetus and placenta. IGF2 can bind to both IGF1 and IGF2 receptors on target cells.

A major target for PLAG1 protein is the *IGF2* gene and PLAG1 binding sites are present in the promoter of *IGF2* (Voz et al., 2000, 2004; Zatkova et al., 2004; Van Dyck et al., 2007; Akhtar et al., 2012; Wang et al., 2013). *IGF2* codes for the IGF2 protein which is an important fetal mitogen (O’Dell & Day, 1998; Curchoe et al., 2005; Berkowicz et al., 2010; Bergman et al., 2013). It is generally accepted that growth in cattle is at least partly associated with variants of *PLAG1*, and differential regulation of *IGF2* by PLAG1 protein (Bolormaa et al., 2015). IGF2 is produced by placental tissue and acts in both the placenta and fetus (Constância et al., 2002; Figure 3). The developing fetus likewise produces IGF2 which acts at the fetus and placenta (Akhtar et al., 2012; Agrogiannis et al., 2014; Sandovici et al., 2022). Inactivation of *PLAG1* is associated with reduced IGF2 and fetal growth retardation (Hensen et al., 2004; Varrault et al., 2006; Habib et al., 2018). Aberrant imprinting of *PLAG1* and overexpression is associated with the large fetus syndrome (Chen et al., 2015). Relationships between *PLAG1*, IGF1 and phenotype have been described for cattle (Fortes et al., 2013).

## *PLAG1*/PLAG1 and Reproduction

### Puberty

Age at puberty is a highly important trait which is linked to lifetime fertility in female cattle (Hawken et al., 2012; Wathes et al., 2014; D’Occhio et al., 2019a). Mutations on chromosome 14 (BTA14), in proximity to *PLAG1*, were reported to be associated with puberty in Zebu (*Bos indicus*) heifers including Brahman (Hawken et al., 2012; Fortes et al., 2013) and Nellore (Mota et al., 2020). Heifers with delayed puberty linked to various *PLAG1* mutations are heavier at puberty. Over 36 yr, we have subjected a herd of Brahman (*Bos indicus*) females to uncompromising selection for fertility (Collins Belah Valley [CBV] Brahman, Belah Valley Cattle Station, Marlborough, Central Queensland, Australia). Females remain in this herd only if they conceive, wean a calf, and reconceive in successive years starting with their first mating (Collins A. Snr., J. E. Kinder, and M. J. D’Occhio, unpublished). Days-to-calving (DTC), defined as the number of days from the start of mating to subsequent calving, is the most important measure of fertility in Brahman and the key driver of profit in beef production. Herd records are used to calculate estimates of genetic differences between animals for DTC and these are expressed as estimated breeding value (EBV) or estimated progeny difference. Female cattle with a low DTC EBV show early puberty as heifers and resume cyclic ovarian function sooner after calving. The DTC EBV for the CBV Brahman herd is −16.8 d compared with the Australian Brahman breed average DTC EBV of −3.2 d. The latter demonstrates a strong genetic component for high fertility of the CBV Brahman herd. It was recently shown that maiden heifers in the CBV Brahman herd that were homozygous for the *PLAG1* variant rs109815800 (G > T) conceived earlier and had greater fertility than contemporary heifers with either one or no copies of the variant (Engle & Hayes, 2022). Heifers with two copies of the variant had a smaller stature than heifers with one or no copies (Engle & Hayes, 2022).

### Ovarian follicles and embryonic and fetal development

**Figure 4.**
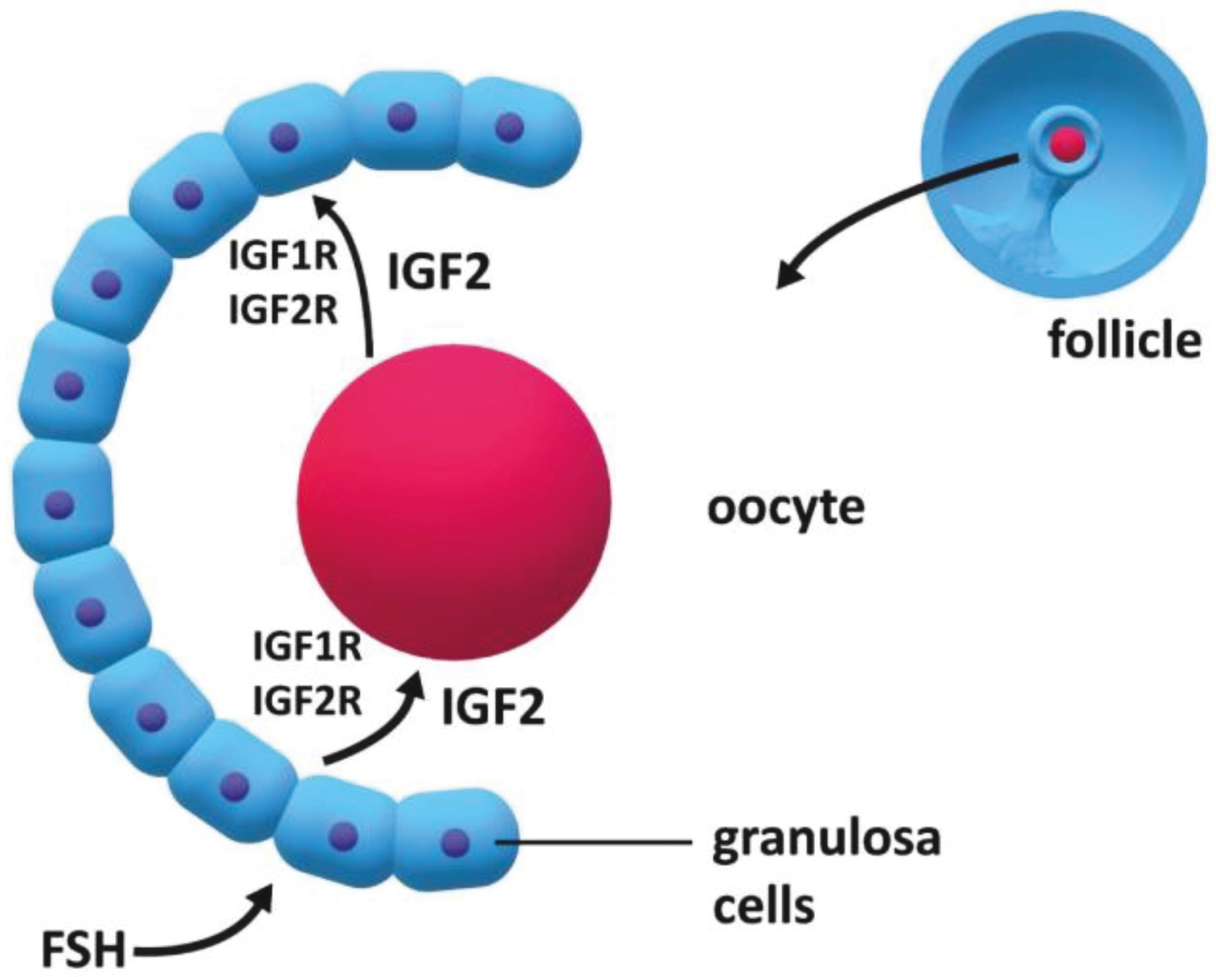
Insulin-like growth factor 2 (IGF2) is produced by oocytes and granulosa cells of follicles and has a local and reciprocal action in oocytes and follicles. IGF2 is an important mitogen and can bind to both IGF1 and IGF2 receptors at target cells. The *IGF2* gene is influenced by PLAG1 protein which provides a mechanism for PLAG1 to be associated with oocyte and follicular function.

**Figure 5.**
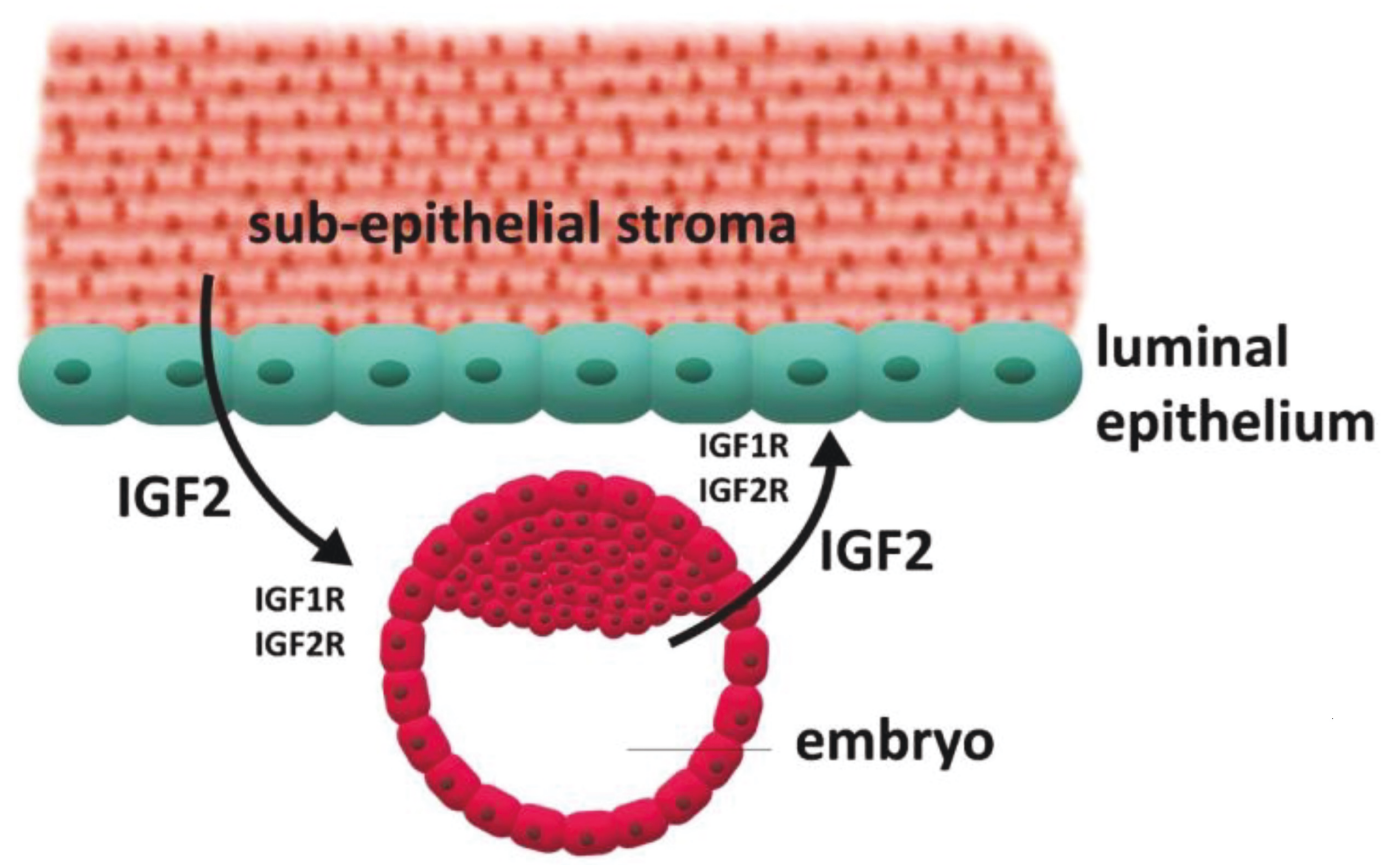
Insulin-like growth factor 2 (IGF2) is produced by the embryo and uterine stroma and has a local and reciprocal action in embryos and uterus. IGF2 is an important mitogen and can bind to both IGF1 and IGF2 receptors at target cells. The *IGF2* gene is influenced by PLAG1 protein which provides a mechanism for PLAG1 to be associated with embryonic and uterine function.

In addition to an effect on age at puberty, *PLAG1*/PLAG1 have been broadly associated with reproductive function in fish and mammals (Pendeville et al., 2006; Juma et al., 2016, 2017, 2018; Wong et al., 2020a, b). The relationship between *PLAG1* and *IGF2* in growth and development, which is discussed above, can be extended to ovarian function and embryonic development in cattle (Neirijnck et al., 2019). *IGF2* is expressed in growing ovarian follicles and has important mitogenic actions on both the follicle and oocyte (Hunter et al., 2004; Spicer & Aad, 2007; Brogan et al., 2010; Aad et al., 2013; Baumgarten et al., 2015; Tkachenko et al., 2021; Figure 4). Oocytes also produce IGF2 which influences the function of oocytes and follicles (Willhelm et al., 2021; Figure 4). IGF2 is additionally expressed by early embryos and the uterus and is involved in autocrine, paracrine, and endocrine events associated with embryonic growth, attachment, and implantation (Robinson et al., 2000; Willhelm et al., 2021; Figure 5). *IGF2* is maternally imprinted similar to *PLAG1* (DeChiara et al., 1991; Giannoukakis et al., 1993; Dindot et al., 2004; Gebert et al., 2006, 2009; Sandovici et al., 2022). As noted above, in early embryos PLAG1 protein acts at the promotor region of over 1,000 genes including IGF2 (Madissoon et al., 2019). Mouse embryos lacking maternal *PLAG1* transitioned slowly from the 2- to 4-cell stage of development (Madissoon et al., 2019). Embryos that transition through early cell divisions in a timely manner have a greater likelihood of surviving and establishing a pregnancy. In mice that lacked maternal *PLAG1* the gene was expressed ectopically from the paternal allele earlier than would otherwise occur (Madissoon et al., 2019).

MicroRNAs (miRNAs) have been implicated in the function of *PLAG1*/PLAG1 in early development (Maccani & Marsit, 2011; Kochhar et al., 2021). For example, miRNA-141 downregulates *PLAG1* translation which is associated with fetal growth retardation (Tang et al., 2013). Based on the relationship between PLAG1 and expression of the *IGF2* gene discussed above, it was concluded that miRNA-141 downregulation of PLAG1 results in reduced IGF2, and suppressed fetal growth (Varrault et al., 2006; Tang et al., 2013; Saha et al., 2015). There is a lack of information on the specific localization of *PLAG1* expression in the embryos and uterus and this is an area that warrants investigation.

## *PLAG1*/PLAG1 and Embryos Survival in Cattle

As noted above, the failure of embryos to progress to implantation and pregnancy is the major cause of reproductive loss in cattle. A deeper understanding of the factors which support embryonic development, attachment, and implantation, is key to improving embryo survival and achieving a transformational increase in reproductive success in female cattle. The factors are both genetic and non-genetic, although these are clearly interrelated. As noted above, Brahman (*Bos indicus*) heifers homozygous for the *PLAG1* variant, rs109815800 (G > T), show greater fertility than contemporary heifers with either one or no copy of the variant. Greater fertility in heifers homozygous for rs109815800 could be due to an earlier age at puberty and/or an increased propensity for embryo survival. The latter would mean that homozygous heifers require fewer matings to achieve pregnancy; typical embryo loss in cattle is in the order of 40% to 60%. Another *PLAG1* variant, rs109231213, appears to be associated with central mechanisms of puberty in heifers (Fortes et al., 2013, 2016; DeAtley et al., 2018). Based on the information provided in this review, it is plausible that *PLAG1*/PLAG1 have a role in embryonic development and survival in cattle. This is supported by the important roles of IGF2 in follicles/oocytes, embryos, and fetuses, and the regulation of *IGF2*/IGF2 by PLAG1. A role in central mechanisms associated with puberty in cattle is also plausible.

Notwithstanding the body of evidence that links *PLAG1*/PLAG1 with IGF2 and embryonic development and reproduction generally, it is noted that some of the relationships in this review could be considered associations and further research is needed to demonstrate additional cause-and-effect relationships.

## Summary

The present review has looked at the emerging roles of *PLAG1*/PLAG1 in embryonic development, placentation, and fetal growth. The most notable expression of *PLAG1* occurs during embryonic and fetal development, with lesser expression after birth. Overexpression of *PLAG1* is associated with the large calf syndrome in cattle and under-expression is linked to fetal growth restriction in cattle and humans. The overexpression of *PLAG1* later in life is typically associated with the formation of solid tumors and cancers. Hence, the expression of *PLAG1* is finely balanced, and disruption in expression at different stages in life shifts *PLAG1* from having beneficial effects to adverse outcomes. *PLAG1*/PLAG1 influence the expression of the *IGF2* gene, and the IGF2 protein is an important mitogen in reproduction. The *PLAG1*-*IGF2* axis, therefore, provides a mechanistic basis for an effect of PLAG1 on ovarian follicles/oocytes, embryos, and fetuses. Our own work involving the selection of Brahman (*Bos indicus*) female cattle for fertility over a period of 35 yr has led to a herd in which heifers homozygous for the *PLAG1* variant, rs109815800, have greater fertility than contemporary heifers with either one or no copy of the variant (Collins A. Snr, J. E. Kinder, B. J. Hayes, and M. J. D’Occhio, unpublished). *PLAG1*/PLAG1 would therefore appear to have important roles in embryonic development and pregnancy in cattle similar to other mammals.

## Abbreviations

CBV: Collins Belah Valley
CHCHD7: coiled-coil-helix-coiled-coil-helix domain containing 7
EBV: estimated breeding value
IGF2: insulin-like growth factor 2
LIF: leukemia inhibitory factor
PLAG1: pleomorphic adenoma gene 1
